# High density lipoprotein particle size and function associate with new cardiovascular events in patients with chronic kidney disease

**DOI:** 10.1371/journal.pone.0320803

**Published:** 2025-04-01

**Authors:** Anna V Mathew, Yun Han, Vetalise C Konje, Yanhong Guo, Jaeman Byun, Alexander George, Julian Meza, Sanjay Rajagopalan, Y. Eugene Chen, Brenda Gillespie, Rajiv Saran, Subramaniam Pennathur

**Affiliations:** 1 Department of Internal Medicine, University of Michigan Medical School, Ann Arbor, Michigan, United States of America; 2 Oakland University William Beaumont School of Medicine, Rochester, Michigan, United States of America; 3 University of Michigan, Ann Arbor, Michigan, United States of America; 4 Department of Medicine, Case Western Reserve University, Cleveland, Ohio, United States of America; 5 School of Public Health, University of Michigan, Ann Arbor, Michigan, United States of America; University Hospital Zurich: UniversitatsSpital Zurich, SWITZERLAND

## Abstract

Chronic Kidney Disease (CKD) is a risk factor for cardiovascular disease (CVD), and patients with CKD have markedly higher CVD mortality compared to healthy controls. However, the relationship between specific lipoprotein profiles and new CV events in patients with advanced CKD and cardiovascular burden is unknown. We profiled the distribution of High density lipoprotein (HDL) size, particle concentration, and cholesterol and triglyceride content of the baseline plasma of 325 subjects with moderate CKD followed for 2.5 years using nuclear magnetic resonance (NMR) spectroscopy. We used Cox regression models controlled for various clinical factors to characterize the role of specific HDL profiles in predicting CV events in this high-risk population. The cholesterol uptake capacity of HDL from peripheral tissues- cholesterol efflux capacity (CEC) and HDL oxidation were also quantified using standardized assays. Patients with new CV events demonstrated increased HDL size, large HDL particle numbers, and CEC. Increased HDL particle size [HR = 2.56, p = 0.002], large HDL particle numbers [HR = 1.41, p = 0.001], HDL-cholesterol levels [HR = 1.03, p = 0.008], and CEC [HR = 1.46, p = 0.03] associated with CV events. Our study demonstrates that higher HDL particle size associated with new CV events in the CKD population with a high cardiovascular burden independent of CEC and HDL cholesterol. Collectively, the data strongly associate altered lipoprotein metabolism, particularly HDL metabolism, and new CV events in patients with established CKD and CVD, allowing us to risk stratify and potentially reduce mortality and morbidity in this high-risk population.

## Introduction

Chronic Kidney Disease (CKD) is prevalent in 14% of US adults, with around 800,000 needing renal replacement therapies like dialysis or renal transplant [1]. Cardiovascular disease (CVD) is the leading cause of morbidity and mortality in CKD, as the odds of CVD are 10-30-fold higher in patients with CKD compared to the age, race, and sex-matched general population [2]. Traditional risk factors like obesity, diabetes, hypertension, and dyslipidemia are associated with this increased CVD risk, in addition to several non-traditional risk factors like inflammation, oxidative stress, uremic toxins, and volume overload [2].

Dyslipidemia in patients with decreased kidney function is characterized by low high density cholesterol (HDL-C) and high triglyceride-rich lipoproteins secondary to downregulated lecithin- cholesterol acyl transferase (LCAT) and, to a lesser extent, increased cholesterol ester transfer protein (CETP) [3]. HDL-C in patients with CKD is not a strong predictor of incident CV events in CKD, unlike the general population [4–7]. Increased total HDL particle concentration has been linked to decreased risk for CVD after controlling for HDL-C in the general population [8–11]. However, in CKD patients, HDL particle concentration was negatively correlated with total CVD but not associated with atherosclerotic CVD and death [12]. In the general population, small HDL particle size levels and reduced mean HDL size increase CV risk [13, 14]. However, large HDL size has also been paradoxically linked to increased CV risk in the EPIC-Norfolk study [15]. Therefore, the association between HDL size and fraction concentrations with CV risk is unclear. CKD patients have lower small, medium, and large HDL particle measures [3,16,17], while dialysis patients had more abundant large HDL fractions than controls [18]. CKD and dialysis patients have altered concentrations in the different HDL fractions, but the exact link between the HDL fractions and CVD risk in moderate to severe CKD patients is completely unknown.

Reduced kidney function alters the HDL proteome, and changes to specific proteins in the HDL proteome are associated with incident CV events in CKD and hemodialysis [19, 20]. Oxidation of HDL proteins by myeloperoxidase (MPO) is linked to HDL dysfunction and predicts CV events and disease in the general population and patients with CKD [21–23]. The primary function of HDL is the transport of cholesterol from foam cells in the vascular wall to their eventual excretion by the liver – also known as reverse cholesterol transport. This HDL function can be measured by a well-described *in vitro* assay called cholesterol efflux capacity (CEC). CEC is related to the prevalence and incidence of CVD independent of HDL cholesterol levels [24, 25]. However, in CKD patients, the association between CEC and CV incident events is absent in several studies. Meanwhile, the role of HDL particle size and concentration in predicting new CV events in moderate to severe CKD- a population with a high prevalence of CVD is unknown. In this study, we characterized the HDL profiles of patients with moderate to severe CKD with a heavy burden of CVD and delineated the risk for new CV events.

## Materials and methods

### Study design

The RRI-CKD (Renal Research Institute) cardiac sub-study was a four-center, prospective, observational cohort study involving adult patients with moderate-to-severe CKD, not on dialysis, enrolled between June 1st, 2000 and February 28^th^, 2006. Eligibility criteria included age ≥  18 years and estimated glomerular filtration rate (eGFR) ≤  50 mL/min by the MDRD formula on two separate occasions. We collected data on demographic characteristics, anthropometric measures, CKD etiology and comorbidities, symptoms, laboratory values, and medications and plasma at enrollment and follow-up visits. The data and samples were accessed on April 1^st^, 2020. The institutional review boards of the participating centers approved the initial study protocol, and written informed consent was obtained at the time of enrollment. A subset of participants underwent specific noninvasive cardiovascular studies, including computed tomography (CT) based Agatston calcium scores, carotid intimal media thickness (CIMT), left ventricular mass index (LVMI), flow-mediated dilation (FMD), and pulse wave velocity (PWV) [26]. Participant follow-up concluded on December 31, 2006, and the median follow-up time was 2.16 years. Study coordinators ascertained all outcomes on an ongoing basis based on regular review of electronic health records, direct patient contact in the clinic, and telephone communication. We considered incident CVD events to include coronary artery disease (acute myocardial infarction, coronary artery bypass grafting, coronary artery stent), cerebrovascular disease (carotid endarterectomy, stroke), peripheral vascular disease (arterial bypass, peripheral artery disease, claudication, chronic extremity ulceration, cellulitis/gangrene), and other CVD (abdominal artery aneurysm, aortic or mitral valve replacement or repair, cardiac arrest, congestive heart failure) [26]. The University of Michigan Institutional Review Board approved the use of the stored specimens and data and waived the need for additional consent.

### Quantification of cholesterol efflux capacity and lipoprotein fractions

Plasma samples (non-fasting) were obtained at baseline (n = 325), stored at -80° C, and analyzed at 15-18 years post-enrollment, depending on the enrollment date. J774 murine macrophages were labeled with 2μCi/mL 3H cholesterol (Perkin Elmer, Waltham, MA) for 24 hours in the presence of ACAT inhibitor (Sandoz 58–035) and equilibrated overnight with 0.3mM 8-(4-chlorophenylthio)-cyclic AMP to induce ABCA1 expression. 2.8% v/v ApoB-depleted serum (containing mature HDL, lipid-poor ApoA1, and HDL remodeling factors) was used as the efflux acceptor for 4 hours. Efflux was quantified via liquid scintillation and expressed as a percentage of total cell 3H-cholesterol content [27].

A subset of these patients underwent lipoprotein profiling using nuclear magnetic resonance spectroscopy (NMR, n = 242) offered as a clinical test by LipoScience Incorporated (Raleigh, North Carolina). Samples for lipoprotein particle analysis were thawed, aliquoted (200 µl), refrozen, and shipped on dry ice. Particle concentrations of HDL of different sizes were calculated from the measured amplitudes of their spectroscopically distinct lipid methyl group NMR signals. Weighted-average lipoprotein particle sizes are derived from the sum of the diameter of each subclass multiplied by its relative mass percentage based on the amplitude of its methyl NMR signal [14].

### Quantification of oxidized amino acids in HDL protein fraction

HDL (d =  1.063–1.210 g/ml) was prepared from plasma (n = 325) using sequential ultracentrifugation [27]. HDL proteins were precipitated and delipidated, and oxidized amino acids were quantified using isotopically labeled internal standards: 13C_6_ tyrosine, 13C_6_ 3-chlorotyrosine, and 13C_6_ 3-nitrotyrosine, via liquid chromatography-electrospray ionization tandem mass spectrometry (LC-ESI-MS/MS) with multiple reaction monitoring and positive ion acquisition mode. This assay’s limit of detection was 0.0001 µM/mol tyrosine.

### Statistical analysis

Descriptive statistics were reported as the mean and standard deviation for continuous variables and counts and percentages for categorical variables. Group differences were assessed by t-test for continuous variables, chi-squared tests, or Fisher exact tests for categorical variables. Oxidized amino acid values below the limit of detection (LOD) were imputed with random values between zero and LOD before log-transformation to the base of two for further analysis. Pearson’s correlation was used to determine relationships between oxidized amino acids, CEC, HDL measures, and subclinical testing measures. Cox proportional hazards models were applied to evaluate associations between markers and time to new CV events. Well-established risk factors for CVD within CKD were selected as covariates for Cox proportional hazards models [4,5,7]. Pearson correlation coefficients were calculated, and false discovery rate correction was applied to account for the multiple testing. Statistical significance was defined as P < 0.05. Data management and statistical analyses were performed using SAS version 9.4.

## Results

### Baseline clinical characteristics and lipoprotein measures

We examined the distribution of baseline demographic characteristics, medications, comorbidities, serum level of CV markers, and measures of subclinical CVD of the entire cohort (n = 325) of patients with advanced CKD (Tables 1 and 2). The demographics and clinical characteristics distribution for the subgroup who underwent NMR spectroscopy had similar patterns and are presented in S1 Table. The relationship of HDL measures with clinical characteristics is summarized in S2 Table. HDL profiles were not associated with various markers of subclinical atherosclerosis like calcium scores, CIMT, LVMI, and PWV (S3 Table).

**Table 1:** Baseline demographics and laboratory characteristics by the development of new cardiovascular (CV) events during study. P-values < 0.05 are bolded.

Variables	Total	No CV event	New CV event	p-value
	n = 325	n = 276	n = 49	
		Mean + SD or n (%)		
Age (years)	60.3 ± 14.8	59.1 ± 14.9	67.1 ± 12.3	** < 0.001**
Female	156 (48.0)	138 (50.0)	18 (36.7)	0.09
**Race**				
Black	57 (17.5)	50 (18.1)	7 (14.3)	0.52
White Others	255 (78.5)	214 (77.5)	41 (83.7)	0.34
	13 (4.0)	12 (4.3)	1 (2.0)	0.45
**Etiology of CKD**				
Diabetes	88 (27.2)	65 (23.6)	23 (46.9)	** < 0.001**
Hypertension	155 (47.8)	127 (46.2)	28 (57.1)	0.16
Glomerulonephritis	88 (27.2)	80 (29.1)	8 (16.3)	0.06
Interstitial Kidney Disease	36 (11.1)	34 (12.4)	2 (4.1)	0.09
Polycystic Kidney Disease	21 (6.5)	21 (7.6)	0 (0.0)	**0.05**
Others	46 (14.2)	37 (13.5)	9 (18.4)	0.36
**Clinical and Lab Characteristics**				
Weight (kg)	84.8 ± 19.8	84.1 ± 19.9	88.7 ± 18.7	0.13
BMI (kg/m2)	29.7 ± 6.648	29.6 ± 6.6	30.2 ± 6.8	0.54
Systolic BP (mmHg)	139.5 ± 23.0	139.2 ± 22.3	140.4 ± 26.8	0.74
Diastolic BP (mmHg)	78.5 ± 12.7	79.3 ± 12.4	74.1 ± 13.6	**0.01**
Heart Rate (bpm)	65.8 ± 10.6	65.6 ± 10.3	66.7 ± 12.3	0.52
Total Cholesterol (mg/dL)	190.9 ± 51.1	193.3 ± 50.2	177.4 ± 54.3	**0.04**
HDL (mg/dL)	42.2 ± 14.4	41.7 ± 13.5	44.9 ± 18.8	0.16
LDL (mg/dL)	107.9 ± 38.9	110.9 ± 38.3	91.4 ± 38.0	** < 0.01**
Triglycerides (mg/dL)	148.4 ± 85.0	149.7 ± 83.3	141.4 ± 94.1	0.53
Serum Albumin (g/dL)	4.00 ± 0.45	4.04 ± 0.44	3.83 ± 0.45	** < 0.01**
CRP (mg/dL)	5.54 ± 9.32	5.12 ± 9.23	7.89 ± 9.60	0.06
Serum Calcium (mg/dL)	9.14 ± 0.70	9.18 ± 0.69	8.93 ± 0.72	**0.02**
Serum Phosphorus (mg/dL)	3.72 ± 0.86	3.66 ± 0.81	4.07 ± 1.03	** < 0.01**
Calcium x phosphorus product	34.01 ± 8.08	33.60 ± 7.72	36.33 ± 9.67	**0.03**
Intact Parathyroid Hormone (pg/mL)	167.8 ± 170.2	163.0 ± 171.3	194.9 ± 162.6	0.23
Hematocrit (%)	36.61 ± 4.72	36.78 ± 4.81	35.67 ± 4.10	0.15
Serum Creatinine (mg/dL)	2.66 ± 1.25	2.60 ± 1.18	3.02 ± 1.55	**0.03**
eGFR (ml/min; MDRD)	28.60 ± 11.74	29.1 ± 11.6	25.9 ± 12.0	0.08
UPCR (g/g creatinine)	722.5 ± 1227.7	691.1 ± 1170.1	892.5 ± 1505.2	0.30
**Medications:**				
Statin	153 (47.1)	126 (45.7)	27 (55.1)	0.22
Diuretic	161 (49.5)	130 (47.1)	31 (63.3)	**0.04**
Calcium channel blocker	143 (44.0)	119 (43.1)	24 (49.0)	0.45
Betablocker	166 (51.1)	137 (49.6)	29 (59.2)	0.22
ACE-I/ARB/Renin inhibitor	221 (87.7)	184 (87.2)	37 (90.2)	0.59
Acetylsalicylic acid	118 (36.3)	95 (34.4)	23 (46.9)	0.09
**CKD stage**				0.34
Stage 2	3 (0.9)	3 (1.1)	0 (0.0)	
Stage 3	135 (41.7)	119 (43.3)	16 (32.7)	
Stage 4	150 (46.3)	125 (45.5)	25 (51.0)	
Stage 5	36 (11.1)	28 (10.2)	8 (16.3)	
**Current tobacco use**	37 (11.4)	33 (12.0)	4 (8.2)	0.44
**Tobacco use history**				0.10
Current tobacco use	37 (11.4)	33 (12.0)	4 (8.2)	
Former tobacco use	111 (34.2)	87 (31.5)	24 (49.0)	
Never	172 (52.9)	151 (54.7)	21 (42.9)	
**History of prior CVD**	125 (38.5)	88 (31.9)	37 (75.5)	** < 0.001**

CVD, cardiovascular disease; CV, cardiovascular; CKD, chronic kidney disease; BP, blood pressure; CRP, C-reactive protein; eGFR, estimated glomerular filtration rate; MDRD, modification of diet in renal disease; UPCR, urine protein to creatinine ratio; BMI, body mass index; ACE-I, angiotensin converting enzyme inhibitor; ARB, angiotensin receptor blockers; HDL, high-density lipoprotein; LDL, low-density lipoprotein

**Table 2:** Baseline subclinical cardiac measures, high-density lipoprotein oxidation measures, and lipoprotein profile measures by the development of new cardiovascular (CV) events during the study. P-values < 0.05 are bolded.

Variables	Total	No CV event	New CV event	p-value
	Mean ^+^ SD or n (%)			
**Subclinical Measures of Atherosclerosis**				
Aorta calcium score	668.7 ± 1573.7 (n = 84)	467.4 ± 1310.2(n = 62)	1235.9 ± 2084.4 (n = 22)	**0.05**
Coronary calcium score	565.8 ± 1193.5(n = 84)	349.3 ± 746.0(n = 62)	1176.0 ± 1866.9(n = 22)	**0.01**
CT Score	438.6 ± 966.6(n = 129)	291.7 ± 590.2(n = 102)	993.6 ± 1685.9(n = 27)	** < 0.001**
Max intima-media thickness (mm)	1.43 ± 0.84(n = 182)	1.29 ± 0.72(n = 147)	2.04 ± 1.01 (n = 35)	** < 0.001**
(ECHO) LV mass index	109.4 ± 37.2(n = 211)	105.7 ± 32.8(n = 175)	127.5 ± 50.5 (n = 36)	** < 0.01**
PWV (m/sec)	8.70 ± 3.12(n = 251)	8.44 ± 3.02(n = 213)	10.2 ± 3.30 (n = 38)	** < 0.01**
**HDL Oxidation Measures**	n = 325	n = 276	n = 49	
3-chlorotyrosine (µM/mM tyrosine)	1.20 ± 3.51	1.20 ± 3.56	1.22 ± 3.31	0.97
o,o’-dityrosine (µM/mM tyrosine)	1.01 ± 2.50	1.03 ± 2.54	0.90 ± 2.30	0.74
3-nitrotyrosine (µM/mM tyrosine)	0.72 ± 2.43	0.74 ± 2.55	0.65 ± 1.60	0.81
CEC (%)	10.47 ± 7.95	10.40 ± 8.32	10.9 ± 5.4	0.69
**HDL Measures**	n = 242	n = 205	n = 37	
Total HDL Particles (µmol/L)	26.02 ± 5.65	26.2 ± 5.5	24.9 ± 6.6	0.20
Large HDL (µmol/L)	4.40 ± 3.24	4.20 ± 3.08	5.52 ± 3.91	**0.02**
Medium HDL (µmol/L)	3.50 ± 3.14	3.65 ± 3.19	2.71 ± 2.74	0.10
Small HDL (µmol/L)	18.12 ± 5.50	18.38 ± 5.28	16.70 ± 6.47	0.09
HDL Size (nm)	8.89 ± 0.50	8.85 ± 0.47	9.12 ± 0.63	** < 0.01**
HDL Cholesterol (mg/dL)	38.0 ± 12.1	37.5 ± 11.2	40.9 ± 16.1	0.12

CV, cardiovascular; HDL, high-density lipoprotein; CT, cardiac computed tomography, LV; left ventricle; PWV, pulse wave velocity; CEC, cholesterol efflux capacity

### Association of HDL measures to cardiovascular outcomes

**Fig 1:**
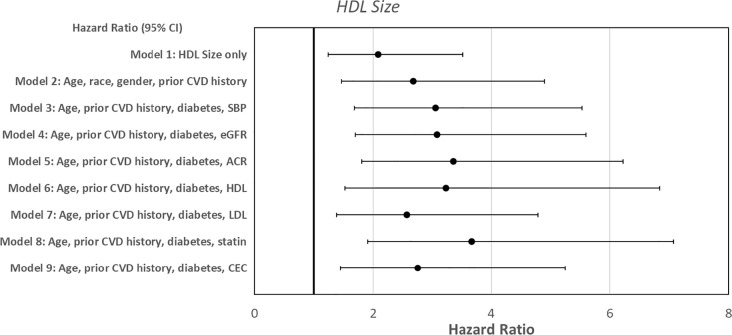
Forest plot depicting Cox proportional models of high-density lipoprotein size predicting increased risk of new cardiovascular events.

**Fig 2:**
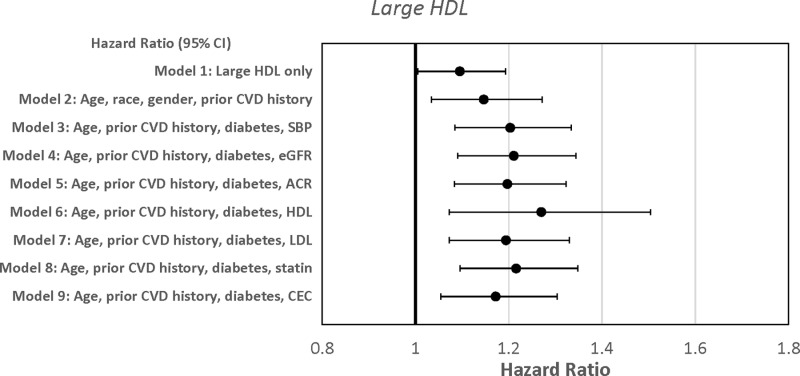
Forest plot depicting the Cox proportional hazard models of large high-density lipoprotein particles predicting time to new cardiovascular events.

Cox proportional hazard models were used to predict the time to first new CVD based on baseline HDL markers. Higher HDL size and large HDL particle numbers were associated with reduced time to CV events (Table 3). In the unadjusted model, a one-unit increase in HDL size at baseline was associated with a 110% higher risk of new CVD (Model 1, HR =  2.09, 95% CI: 1.24, 3.52, p = 0.006). Subsequent models with adjustments for various clinical variables were represented in Fig 1 and Table 3. Each nanomole/liter higher large HDL was associated with a 9.5% higher risk for new CVD (Model 1, HR =  1.09, 95% CI: 1.00, 1.19, p = 0.04) that persisted with adjustment with clinical variables (Fig 2 and Table 3). Adding C-reactive protein (CRP) into the Cox models did not alter the relationship between HDL size and large HDL particles with time to CV events. Total HDL particles, small and medium HDL particles were not associated with time to CV events. In contrast, HDL-C was associated with decreased time to CV events after adjustment for age, history of CVD, diabetes, and BP status, but not in the univariate analysis (HR = 1.04, 95% CI: 1.01, 1.06, p = 0.003) (S4 Table).

**Table 3:** Cox proportional hazards model for time to new cardiovascular event by significant lipoprotein measures. Hazard ratios, 95% confidence intervals, and p-values are displayed. Statistically significant (p < 0.05) hazard ratios and p-values are bolded.

	Model 1		Model 2		Model 3		Model 4		Model 5		Model 6		Model 7		Model 8		Model 9	
	*Univariable model*		*Measure, age, race, gender, prior CVD history*		*Measure, age, prior CVD history, diabetes, SBP*		*Measure, age, prior CVD history, diabetes, eGFR*		*Measure, age, prior CVD history, diabetes, ACR*		*Measure, age, prior CVD history, diabetes, HDL-C*		*Measure, age, prior CVD history, diabetes, LDL-C*		*Measure, age, prior CVD history, diabetes, statin*		Measure, age, prior CVD history, diabetes, CRP	
**Measures** ^*^	**HR**	**p-value**	**HR**	**p-value**	**HR**	**p-value**	**HR**	**p-value**	**HR**	**p-value**	**HR**	**p-value**	**HR**	**p-value**	**HR**	**p-value**	**HR**	**p-value**
	**(95% CI)**		**(95% CI)**		**(95% CI)**		**(95% CI)**		**(95% CI)**		**(95% CI)**		**(95% CI)**		**(95% CI)**		**(95% CI)**	
**Large HDL**	**1.10**	**0.04**	**1.15**	**0.01**	**1.20**	**<.01**	**1.21**	**<.01**	**1.20**	**<.01**	**1.27**	**0.01**	**1.19**	**<.01**	**1.22**	**<.01**	**1.19**	**<.01**
**(µmol/L)**	**(1.01, 1.19)**		**(1.03, 1.27)**		**(1.09, 1.33)**		**(1.09, 1.34)**		**(1.08, 1.32)**		**(1.07, 1.50)**		**(1.07, 1.33)**		**(1.10, 1.35)**		**(1.08,1.32)**	
**HDL Size**	**2.09**	**0.01**	**2.68**	**<.01**	**3.05**	**<.01**	**3.08**	**<.01**	**3.35**	**<.01**	**3.23**	**<.01**	**2.57**	**<.01**	**3.68**	**<.01**	**3.35**	**<.01**
**(nm)**	**(1.24, 3.52)**		**(1.47, 4.90)**		**(1.69, 5.53)**		**(1.70, 5.59)**		**(1.81, 6.22)**		**(1.52, 6.84)**		**(1.38, 4.79)**		**(1.91, 7.08)**		**(1.8,6.21)**	
**HDL Cholesterol**	1.02	0.18	1.03	0.08	**1.04**	**<.01**	**1.04**	**<.01**	**1.04**	**<.01**	**1.09**	**0.03**	**1.03**	**0.01**	**1.04**	**<.01**	**1.03**	**<.01**
**(mg/dL)**	(0.99, 1.04)		(1.00, 1.05)		**(1.01, 1.06)**		**(1.02, 1.07)**		**(1.01, 1.06)**		**(1.01, 1.18)**		**(1.01, 1.06)**		**(1.01, 1.07)**		**(1.01,1.065)**	
**CEC**	1.33	0.15	**1.48**	**0.04**	1.43	0.06	**1.50**	**0.02**	**1.55**	**0.02**	1.41	0.07	**1.43**	**0.05**	**1.50**	**0.04**	**1.49**	**0.02**
**(%)**	(0.90, 1.96)		**(0.02, 2.15)**		(0.99, 2.05)		**(1.06, 2.12)**		**(1.08, 2.22)**		(0.98, 2.03)		**(1.01, 2.05)**		**(1.03, 2.18)**		**(1.05,2.13)**	

* Model 1 is a univariable model with each separate HDL measure, while Models 2-8 represent multivariable models of the respective HDL measures with additional covariates.

CVD, cardiovascular disease; SBP, systolic blood pressure; ACR, albumin to creatinine ratio; HDL-C, high-density lipoprotein cholesterol; LDL-C, low-density lipoprotein cholesterol; CEC, cholesterol efflux capacity; HR, hazard ratio; CI, confidence interval

### Association of HDL function and oxidation markers with cardiovascular outcomes

When looking at the relationship between HDL lipoprotein measures and CEC– CEC was related to HDL particle number, HDL size, large HDL particle, and HDL cholesterol levels, as is to be expected, but not with small or medium size HDL particles (S4 Table). HDL oxidation markers were not correlated with CEC (S6 Table), except for *o,o*′-dityrosine, which correlated with CEC (p = 0.003). The relationship of HDL markers with clinical characteristics is summarized in S7 Table. HDL oxidation markers were not associated with various markers of subclinical atherosclerosis, like calcium scores, CIMT, LVMI, and PWV (S8 Table).

**Fig 3:**
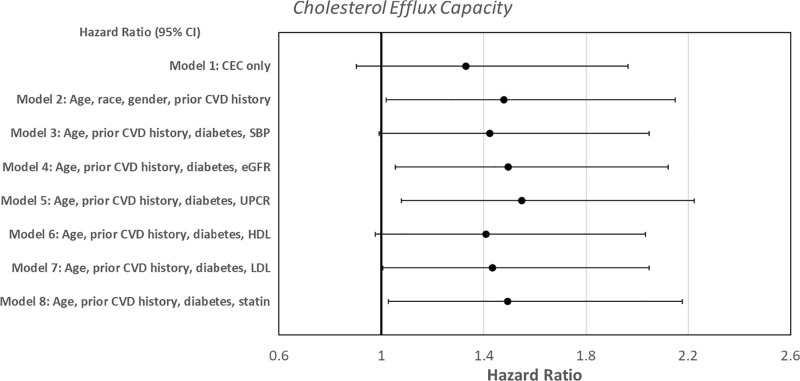
Forest plot depicting the Cox proportional hazard models of cholesterol efflux capacity predicting time to new cardiovascular events.

Cox proportional hazard models were used to predict the time to first new CV events based on baseline CEC and HDL oxidation markers. CEC, a measure of reverse cholesterol transport to HDL fractions, was associated with decreased time to first CV event after adjustment for age, race, and prior history of CVD history (Model 2, HR =  1.48, 95% CI: 0.02, 2.15, p = 0.04, Fig 3 and Table 3) but not in the univariate analyses and specific multivariate models. The addition of CEC did not alter the Cox models of HDL size and large HDL in predicting new CV events represented in Fig 1 and Fig 2, respectively. Similarly, adding CRP into the Cox models did not alter the relationship between CEC and CV events. HDL oxidation markers were not linked to time to CV events (S9 Table).

## Discussion

Our study is the first to report the association between NMR-measured HDL profiles and new CV events in patients with advanced CKD with a high prevalence of well-managed CVD. Specifically, we demonstrate that HDL particle size is associated with new CV events in CKD even when controlled for HDL-C and CEC. In addition, we find that increased CEC, HDL-C, and large HDL particle numbers are similarly linked to decreased time to new CV events in CKD. These findings demonstrate that increased HDL size, either due to increased cholesterol flux into HDL particles or possibly decreased HDL clearance, is linked to new CV events in patients with advanced CKD with established CVD.

Low HDL-C levels are associated with increased CV events and CVD in the general population, whereas raising the HDL-C greater than 80 mg/dL confers an increased CV risk [28]. The prognostic significance of HDL-C in CKD and dialysis populations is even more controversial [4–7]. HDL-C demonstrated a similar U-shaped association in the dialysis population [29], while other CKD and dialysis cohorts had no link between HDL-C and CVD or CV mortality [30–32]. More recent evidence supports a role for HDL class heterogeneity, indicating that specific HDL subclasses are more relevant to CV risk than others. While one study demonstrated higher HDL_2_ cholesterol levels in dialysis patients [33], another study demonstrated a pattern of lower HDL_2_ and higher HDL_3_ in CKD and dialysis patients [34, 35], which was linked with CV events in patients on hemodialysis [36]. Patients with CKD and dialysis have increased smaller sized pre-β HDL compared to larger sized α-HDL [37]– perhaps related to increased CETP activity and decreased renal clearance of lipid-poor HDL and lecithin-cholesterol acyltransferase (LCAT) activity observed in CKD [33]. We find that increased HDL size or larger lipid-rich HDL fractions predict new CV events in our CKD cohort. Our cohort did not include dialysis patients and specifically focused on atherosclerotic events, and these differences might be the reason for these discrepancies.

**Fig 4:**
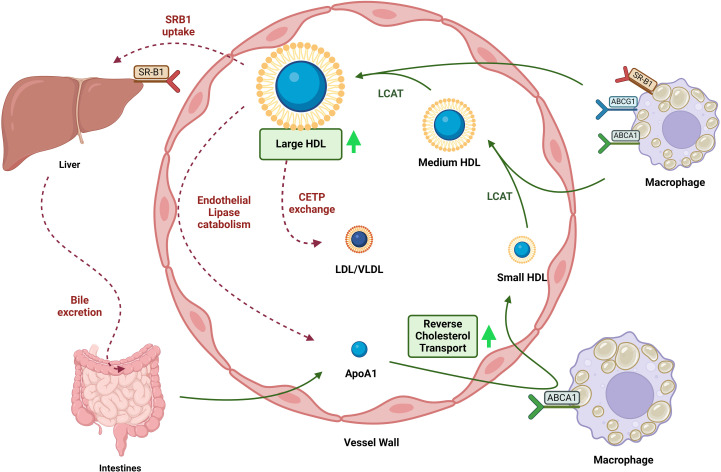
HDL lifecycle. Apolipoprotein A1 (ApoA1), produced by the liver and the intestine, acquires phospholipids (PL) and free cholesterol (FC), generating discoidal pre-β-HDL. Pre-β-HDL particles uptake FC and PL from cells through the reverse cholesterol transport process via the ATP-binding cassette transporters A1/G1 (ABCA1/ABCG1) and scavenger receptor class B type 1 (SR-BI). As the Lecithin-Cholesterol-Acyl- Transfer (LCAT) enzyme esterifies FC within HDL, the nascent discoidal pre-β-HDL becomes quasi-spherical, giving rise to small, medium, and large HDL, the primary form of circulating HDL. In addition to LCAT, phospholipid transfer protein (PLTP) and Cholesterol Ester Transfer Protein (CETP) contribute to HDL remodeling, by the removal of cholesteryl ester (CE) and glycerolipid contents, in exchange for TG with triglyceride-rich lipoproteins (TGRLs). Larger HDL fractions are remodeled by peripheral and hepatic lipases and scavenged by the liver to be excreted as bile.

Increased total HDL particle concentration has been linked to decreased risk for CVD after controlling for HDL-C in the general population [8–11]. Large HDL particle concentration is typically inversely related to CV risk, while small HDL particle size levels were positively related to CV risk [13]. Reduced mean HDL size is linked to CVD in large studies in the general population [14]. Therapeutic interventions like niacin and CETP inhibitors raise HDL-C and preferentially increase large HDL levels raising the average HDL size [38, 39]. Large HDL size has been paradoxically linked to increased CV risk after multiple therapies in the EPIC-Norfolk study [15]. Large HDL particles, which are known to be cardioprotective, are decreased in CKD [3,17]. CKD patients in a Mexican cohort have lower medium and small HDL particle measures [16], while dialysis patients had more abundant large HDL fractions than controls [18]. Meanwhile, the Dallas Heart Study with 210 CKD patients with no history of CVD showed a negative correlation between HDL particle concentration by NMR with total CVD but not atherosclerotic CVD and death [12]. In a more recent study, reduced cholesterol and ApoA I and II content in the small and extra small HDL fractions were linked to all-cause mortality in CKD patients but not to cardiovascular death [40]. This contrasts with our finding that large HDL particle concentration and increased HDL size are linked to atherosclerotic CV events. Plasma-advanced oxidation products [41] and hepatic scavenger receptor-B1 (SRB1) suppression affect the clearance of HDL-C in CKD [42] and could explain the increased large HDL particle concentrations and HDL size linked to increased CV events in our study, similar to patients on dialysis [33]. Our study shows that large HDL particle number and HDL size were independently related to CEC (Table 3), highlighting the increased flux into the reverse cholesterol transport that increases HDL size and large HDL particle numbers as a potential mechanism. We hypothesize that there is an accumulation of these mature large HDL particles either because of the increased flux or decrease in the different mechanisms of clearance of large HDL particles, i.e., SRB1-based removal of HDL-C into bile, phospholipid transfer protein (PLTP) transfer of phospholipids to other particles, and CETP mediated lipid exchange with triglyceride-rich lipoproteins [43] (Fig 4). However, the continued increase in CEC and large HDL particle concentration and size indicate other HDL-related mechanisms like liver clearance are worsening and will eventually impair CEC. Since the association with HDL size and large HDL particle numbers remains intact after adjustment for both HDL-C and CEC, indicating other mechanisms like clearance might be in play, as demonstrated by an earlier study [42].

Patients with CKD demonstrated increased CEC compared to healthy controls and patients with CVD risk and established CVD [42]. Meanwhile, the ability to deliver cholesterol from macrophages to primary hepatocytes was diminished in CKD patients, similar to patients with CVD risk and established CVD, compared to healthy controls, even when corrected for CEC [42]. The Dallas Heart Study with CKD patients with no prior history of CVD showed that increased CEC was associated with increased atherovascular CV risk and death, like our study [12]. In the CARE FOR HOMe study, European CKD patients demonstrated no association between CEC and CV events and all-cause mortality [30], similar to a hemodialysis sub-cohort with type 2 diabetes [44]. In a recent study by our group, no associations with HDL cholesterol or CEC were found in two cohorts with milder CKD. In this study, which used a differential mobility analyzer and mass spectrometry-based classification into six HDL sub-groups based on size from ultracentrifuge-based HDL separation, the medium-sized HDL-P (~9.1 nm) which actually falls in the range of our large HDL in our study (8.8-13 nm), was negatively associated with incident CVD in both cohorts after adjusting for clinical confounders [45]. Our study was performed using clinically certified NMR-based methods from plasma, and the medium-sized HDL in our study showed a similar trend but did not reach significance. The CEC of the individual HDL fractions and enzyme activity influencing HDL degradation would provide invaluable insight into the influence of CEC and HDL clearance on HDL size but is unavailable in this cohort due to sample limitations. Our study demonstrates that increased CEC positively correlates with decreased time to incident CV events in patients with moderate to severe CKD in specific multivariate models but not in the univariate analyses. The association of upregulated CEC, increased HDL size, and large HDL particle concentration with CV events demonstrated in our study strengthens the hypothesis that altered HDL metabolism contributes to atherosclerosis in CKD. Statin use had minimal effect on the regression models and time to CV events in our cohort because of the high statin use in the cohort in both the event and non-event cohorts and perhaps reflects the minimal effect of statin in patients with advanced CKD. Multiple studies have reported that CKD alters the HDL proteome, while our recent study identified HDL proteins associated with new CVD in CKD patients [19, 20]. Similarly, post-translational modifications by enzymes like MPO caused functional changes in HDL proteome predicting CVD in the general population and are associated with CVD in CKD [23]. Our study reveals no association between HDL 3-chlorotyrosine – a specific MPO byproduct and the incidence of CV events.

The current study links HDL profiles to CVD and events in CKD. Our study measured CEC, lipoprotein profiles, and HDL oxidation using well-validated J77 macrophages, NMR, and LCMS assays. Our current study has several limitations. This study is limited in its generalizability due to the recruitment geographical and healthcare settings. This study had an event probability of 15.1% is consistent with other studies of CV events in CKD patients. However, higher event rates would have increased the power over all the measured variables. Further studies, such as the measurement of HDL remodeling factors and the efflux of individual HDL size classes were limited by sample availability. Apo B depleted serum includes mature HDL, free apo A1, and HDL modeling enzymes like CETP, PLTP, and LCAT, which could influence our associations with CEC. As a prospective observational study; we cannot ascribe a causal link between CEC and lipoprotein profiles to the development of CVD within the CKD population. Our study uses the MDRD formula to estimate GFR due to the standard of care at the time of enrollment and formed the basis of diagnosis and treatment of these patients. Recalculating eGFR using the CKD-EPI 2021 formula would require a comprehensive re-analysis of our data, potentially introducing variations that could affect the integrity and comparability of our study outcomes. Changes to HDL profiles parallel the changes to CEC, but multiple alterations in HDL fractions, both in quantity and quality, suggest a more robust and specific link. More experimental studies are needed to elucidate the mechanistic role of the CEC and HDL fractions in the generation of atherosclerotic lesions leading to CVD in CKD. We had to limit the number of covariates in the regression models because of the small number of CV events. Another limitation of our work is the sole evaluation of lipid profiles at enrollment, which prevented time-varying risk association with CVD in our population. A more extensive study with longer follow-up and regular sampling will better investigate the role of these biomarkers in predicting incident CVD within the CKD population.

In conclusion, we demonstrate the role of HDL size, large HDL particle size, and CEC associated with new CV events in patients with moderate to severe CKD.

## Supporting information

S1 Table Baseline demographics and laboratory characteristics of patients with baseline lipoprotein profiles by development of new cardiovascular events.
P-values < 0.05 are bolded.
(DOCX)

S2 Table Correlation of Lipoprotein measures with clinical measures.
Correlation coefficients (r) and corresponding raw p-values are given; significant P-values < 0.05 are indicated with an asterisk *, and those that pass significance after false discovery rate correction are bolded. N = 242.
(DOCX)

S3 Table Relationship of subclinical markers of cardiovascular disease to lipoprotein measures.
Correlation coefficients (r) and corresponding raw p-values are given; significant P-values < 0.05 are indicated with an asterisk *, and those that pass significance after false discovery rate correction are bolded.
(DOCX)

S4 Table Cox proportional hazards model for time to the new cardiovascular event by significant high-density lipoprotein measures.
Hazard ratios, 95% confidence intervals, and p-values are displayed. Statistically significant (p < 0.05) hazard ratios and p-values are bolded.
(DOCX)

S5 Table Relationship of high-density lipoprotein measures to cholesterol efflux.
Correlation coefficients (r) and corresponding raw p-values are given; significant P-values < 0.05 are indicated with an asterisk *, and those that pass significance after false discovery rate correction are bolded. N = 242.
(DOCX)

S6 Table Relationship of high-density lipoprotein measures to high-density lipoprotein oxidation measures.
Correlation coefficients (r) and corresponding raw p-values are given; significant P-values < 0.05 are indicated with an asterisk *, and those that pass significance after false discovery rate correction are bolded. N = 242.
(DOCX)

S7 Table Correlation of cholesterol efflux capacity and high-density lipoprotein oxidation measures with clinical measures.
Correlation coefficients (r) and corresponding raw p-values are given; significant P-values < 0.05 are indicated with an asterisk *, and those that pass significance after false discovery rate correction are bolded. N = 325.
(DOCX)

S8 Table Relationship of lipoprotein oxidation measures with subclinical markers of cardiovascular disease.
Correlation coefficients (r) and corresponding raw p-values are given; significant P-values < 0.05 are indicated with an asterisk *, and those that pass significance after false discovery rate correction are bolded.
(DOCX)

S9 Table Cox proportional hazards model for time to the new cardiovascular event by significant lipoprotein oxidation measures.
Hazard ratios, 95% confidence intervals, and p-values are displayed. Statistically significant (p < 0.05) hazard ratios and p-values are bolded.
(DOCX)
