# Supplementary material for: High density lipoprotein particle size and function associate with new cardiovascular events in patients with chronic kidney disease
Source: PLoS One. 2025 Apr 1;20(4):e0320803. doi: 10.1371/journal.pone.0320803 (PMC11960887; doi:10.1371/journal.pone.0320803)
Supplement: S1 Table — P-values < 0.05 are bolded. (DOCX) [file pone.0320803.s001.docx]

| **S1 Table. Baseline demographics and laboratory characteristics of patients with baseline lipoprotein profiles by development of new cardiovascular events.** P-values <0.05 are bolded. | | | | | |
| --- | --- | --- | --- | --- | --- |
| **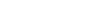Variables** | | **Total** | **No CV event** | **New CV event** | **p-value** |
|  |  | n=242 | n=205 | n=37 |  |
|  |  | Mean + SD or n (%) | | |  |
| Age (years) | | 59.5 ± 14.8 | 58.4 ± 14.9 | 65.5 ± 12.8 | **0.01** |
| Female | | 113 (46.7) | 99 (48.3) | 14 (37.8) | 0.24 |
| **Race** | |  |  |  |  |
|  | Black | 40 (16.5) | 35 (17.1) | 5 (13.5) | 0.59 |
|  | White | 189 (78.1) | 158 (77.1) | 31 (83.8) | 0.36 |
|  | Others | 13 (5.4) | 12 (5.9) | 1 (2.7) | 0.43 |
| **Etiology of CKD** | |  |  |  |  |
|  | Diabetes | 60 (24.9) | 45 (22.1) | 15 (40.5) | **0.02** |
|  | Hypertension | 106 (44.0) | 88 (43.1) | 18 (48.6) | 0.53 |
|  | Glomerulonephritis | 72 (29.9) | 66 (32.4) | 6 (16.2) | **0.05** |
|  | Interstitial Renal Disease | 28 (11.6) | 27 (13.2) | 1 (2.7) | 0.07 |
|  | Polycystic Kidney Disease | 17 (7.1) | 17 (8.3) | 0 (0.0) | 0.07 |
|  | Others | 32 (13.3) | 24 (11.8) | 8 (21.6) | 0.10 |
| **Clinical and Lab Characteristics** | |  |  |  |  |
|  | Weight (kg) | 84.1 ± 18.8 | 83.6 ± 19.2 | 86.5 ± 16.5 | 0.40 |
|  | BMI (kg/m2) | 29.4 ± 6.4 | 29.4 ± 6.3 | 29.4 ± 7.0 | 0.98 |
|  | Systolic BP (mmHg) | 139.8 ± 24.1 | 139.6 ± 23.2 | 140.8 ± 28.8 | 0.78 |
|  | Diastolic BP (mmHg) | 78.7 ± 12.4 | 79.3 ± 11.9 | 75.4 ± 14.6 | 0.08 |
|  | Heart Rate (bpm) | 66.4 ± 10.6 | 66.6 ± 10.6 | 65.4 ± 10.5 | 0.50 |
|  | Total Cholesterol (mg/dL) | 193.7 ± 52.5 | 197.0 ± 50.9 | 175.3 ± 57.8 | **0.02** |
|  | HDL (mg/dL) | 40.2 ± 12.5 | 39.9 ± 11.9 | 41.6 ± 15.5 | 0.46 |
|  | LDL (mg/dL) | 111.4 ± 39.5 | 114.8 ± 38.4 | 93.0 ± 40.8 | **<0.01** |
|  | Triglycerides (mg/dL) | 140.8 ± 73.8 | 144.0 ± 74.0 | 123.4 ± 71.0 | 0.12 |
|  | Serum Albumin (g/dL) | 4.01 ± 0.45 | 4.04 ± 0.45 | 3.85 ± 0.46 | **0.02** |
|  | CRP (mg/dL) | 6.02 ± 10.28 | 5.62 ± 10.38 | 8.24 ± 9.50 | 0.16 |
|  | Serum Calcium (mg/dL) | 9.17 ± 0.66 | 9.23 ± 0.65 | 8.88 ± 0.66 | **<0.01** |
|  | Serum Phosphorus (mg/dL) | 3.74 ± 0.92 | 3.67 ± 0.86 | 4.14 ± 1.14 | **<0.01** |
|  | Intact Parathyroid Hormone (pg/mL) | 178.5 ± 181.3 | 172.2 ± 183.0 | 212.9 ± 170.0 | 0.21 |
|  | Hematocrit (%) | 36.5 ± 4.5 | 36.7 ± 4.5 | 35.3 ± 4.4 | 0.12 |
|  | Serum Creatinine (mg/dL) | 2.84 ± 1.32 | 2.76 ± 1.25 | 3.30 ± 1.58 | **0.02** |
|  | eGFR (ml/min; MDRD) | 26.5 ± 10.8 | 27.2 ± 10.9 | 22.5 ± 9.3 | **0.01** |
|  | UPCR (g/g creatinine) | 790.9 ± 1325.8 | 749.0 ± 1257.5 | 1015.1 ± 1646.4 | 0.26 |
| **Medications:** | |  |  |  |  |
|  | Statin | 111 (45.9) | 90 (43.9) | 21 (56.8) | 0.15 |
|  | Diuretic | 116 (47.9) | 92 (44.9) | 24 (64.9) | **0.03** |
|  | Calcium channel blocker | 109 (45.0) | 90 (43.9) | 19 (51.4) | 0.40 |
|  | Betablocker | 121 (50.0) | 95 (46.3) | 26 (70.3) | **<0.01** |
|  | ACE-I/ARB/Renin inhibitor | 169 (86.2) | 139 (85.8) | 30 (88.2) | 0.71 |
|  | Acetylsalicylic acid | 87 (36.0) | 70 (34.1) | 17 (45.9) | 0.17 |
| **CKD stage** | |  |  |  | 0.20 |
|  | Stage 2 | 1 (0.4) | 1 (0.5) | 0 (0.0) |  |
|  | Stage 3 | 81 (33.6) | 74 (36.3) | 7 (18.9) |  |
|  | Stage 4 | 126 (52.3) | 103 (50.5) | 23 (62.2) |  |
|  | Stage 5 | 33 (13.7) | 26 (12.7) | 7 (18.9) |  |
| **Current tobacco use** | | 28 (11.6) | 26 (12.7) | 2 (5.4) | 0.20 |
| **Tobacco use history** | |  |  |  | 0.07 |
|  | Current tobacco use | 28 (11.6) | 26 (12.7) | 2 (5.4) |  |
|  | Former tobacco use | 76 (31.4) | 58 (28.3) | 18 (48.6) |  |
|  | Never | 133 (55.0) | 116 (56.6) | 17 (45.9) |  |
| **History of prior CVD** | | 88 (36.4) | 59 (28.8) | 29 (78.4) | **<0.01** |
| **Oxidation Measures** | |  |  |  |  |
|  | 3-chlorotyrosine (µM/mM tyrosine) | 1.38 ± 3.83 | 1.46 ± 4.06 | 0.92 ± 2.20 | 0.43 |
|  | o,o'-dityrosine (µM/mM tyrosine) | 1.14 ± 2.86 | 1.19 ± 2.91 | 0.88 ± 2.92 | 0.55 |
|  | 3-nitrotyrosine (µM/mM tyrosine) | 0.88 ± 2.78 | 0.89 ± 2.92 | 0.79 ± 1.81 | 0.83 |
|  | CEC (%) | 10.0 ± 5.67 | 9.83 ± 5.65 | 10.95 ± 5.75 | 0.28 |
| **Subclinical Measures** | |  | |  | |
|  | Aorta calcium score | 683.9 ± 1599.3 (n-81) | 462.3 ± 1320.5 (n=61) | 1359.5 ± 2150.6 (n=20) | **0.03** |
|  | Coronary calcium score | 519.9 ± 1183.4 (n=81) | 324.5 ± 726.0 (n=61) | 1115.8 ± 1932.8 (n=20) | **0.01** |
|  | CT Score | 466.1 ± 1060.3 (n=99) | 319.9 ± 640.15 (n=77) | 977.5 ± 1846.9 (n=22) | **0.01** |
|  | Max intima-media thickness (mm) | 1.41 ± 0.84 (n=136) | 1.29 ± 0.72 (n=110) | 1.98 ± 1.03 (n=26) | **< 0.01** |
|  | (ECHO) LV mass index | 109.25 ± 37.2 (n=203) | 105.5 ± 32.3 (n=169) | 128.0 ± 51.9 (n=34) | **0.001** |
|  | PWV (m/sec) | 8.8 ± 3.12 (n=185) | 8.6 ± 3.04 (n=156) | 10.3 ± 3.65 (n=29) | **< 0.01** |
| CV, cardiovascular disease; CV, cardiovascular; CKD, chronic kidney disease; BP, blood pressure; CRP, C-reactive protein; eGFR, estimated glomerular filtration rate; MDRD, modification of diet in renal disease; UPCR, urine protein to creatinine ratio; BMI, body mass index; ACE-I, angiotensin converting enzyme inhibitor; ARB, angiotensin receptor blockers; HDL, high-density lipoprotein; LDL, low-density lipoprotein | | | | | |
