# Supplementary material for: High density lipoprotein particle size and function associate with new cardiovascular events in patients with chronic kidney disease
Source: PLoS One. 2025 Apr 1;20(4):e0320803. doi: 10.1371/journal.pone.0320803 (PMC11960887; doi:10.1371/journal.pone.0320803)
Supplement: S2 Table — Correlation coefficients (r) and corresponding raw p-values are given; significant P-values < 0.05 are indicated with an asterisk *, and those that pass significance after false discovery rate correction are bolded. N = 242. (DOCX) [file pone.0320803.s002.docx]

| **S2 Table. Correlation of Lipoprotein measures with clinical measures.** Correlation coefficients (r) and corresponding raw p-values are given; significant P-values <0.05 are indicated with an asterisk*, and those that pass significance after false discovery rate correction are bolded. N=242. | | | | | | | | | | | | | | | | | | | | | | | | | | | | | | | | |
| --- | --- | --- | --- | --- | --- | --- | --- | --- | --- | --- | --- | --- | --- | --- | --- | --- | --- | --- | --- | --- | --- | --- | --- | --- | --- | --- | --- | --- | --- | --- | --- | --- |
|  | **Blood Pressure (mmHg)** | | | | | **CRP (mg/dL)** | | | **eGFR (ml/min)** | | | | **UPCR**  **(g/g creatinine)** | | | | **Serum Albumin (g/dL)** | | | | **Cholesterol (mg/dL)** | | | | **HDL (mg/dL)** | | | | **LDL (mg/dL)** | | |  |
| **Measures** | **r** | | | **p-value** | | **r** | **p-value** | | **r** | | **p-value** | | **r** | | **p-value** | | **r** | | **p-value** | | **r** | | **p-value** | | **r** | | **p-value** | | **r** | **p-value** | |  |
| **Total HDL Particles (µmol/L)** | 0.04 | 0.57 | ***-0.17*** | | ***0.01*** | | | 0.09 | | 0.17 | | 0.08 | | 0.24 | | 0.13* | | 0.05* | | ***0.35*** | | ***<.01*** | | ***0.58*** | | ***<.01*** | | ***0.15*** | | | ***0.02*** |  |
| **Large HDL (µmol/L)** | 0.01 | 0.88 | -0.02 | | 0.73 | | | -0.06 | | 0.32 | | 0.04 | | 0.53 | | ***-0.21*** | | ***<.01*** | | 0.03 | | 0.69 | | ***0.78*** | | ***<.01*** | | -0.13* | | | 0.04* |  |
| **Medium HDL (µmol/L)** | -0.05 | 0.41 | 0.10 | | 0.12 | | | 0.05 | | 0.43 | | -0.11 | | 0.09 | | 0.11 | | 0.08 | | 0.14* | | 0.03* | | -0.08 | | 0.24 | | 0.09 | | | 0.18 |  |
| **Small HDL (µmol/L)** | 0.06 | 0.33 | ***-0.22*** | | ***<.01*** | | | 0.10 | | 0.12 | | 0.12 | | 0.07 | | ***0.19*** | | ***<.01*** | | ***0.26*** | | ***<.01*** | | ***0.18*** | | ***0.01*** | | ***0.18*** | | | ***<.01*** |  |
| **HDL Size (nm)** | 0.03 | 0.67 | 0.02 | | 0.73 | | | -0.09 | | 0.17 | | -0.02 | | 0.81 | | ***-0.22*** | | ***<.01*** | | -0.04 | | 0.50 | | ***0.65*** | | ***<.01*** | | ***-0.19*** | | | ***<.01*** |  |
| **HDL cholesterol (mg/dL)** | 0.01 | 0.84 | -0.10 | | 0.13 | | | 0.00 | | 0.95 | | 0.06 | | 0.38 | | -0.09 | | 0.18 | | ***0.21*** | | ***<.01*** | | ***0.90*** | | ***<.01*** | | -0.04 | | | 0.50 |  |
| eGFR, estimated glomerular filtration rate; HDL, high-density lipoprotein; UPCR, urine protein creatinine ratio; CRP, C-reactive protein | | | | | | | | | | | | | | | | | | | | | | | | | | | | | | | | |
