# Supplementary material for: High density lipoprotein particle size and function associate with new cardiovascular events in patients with chronic kidney disease
Source: PLoS One. 2025 Apr 1;20(4):e0320803. doi: 10.1371/journal.pone.0320803 (PMC11960887; doi:10.1371/journal.pone.0320803)
Supplement: S3 Table — Correlation coefficients (r) and corresponding raw p-values are given; significant P-values < 0.05 are indicated with an asterisk *, and those that pass significance after false discovery rate correction are bolded. (DOCX) [file pone.0320803.s003.docx]

| **S3 Table. Relationship of subclinical markers of cardiovascular disease to lipoprotein measures.** Correlation coefficients (r) and corresponding raw p-values are given; significant P-values <0.05 are indicated with an asterisk*, and those that pass significance after false discovery rate correction are bolded. | | | | | | | | | | | | |
| --- | --- | --- | --- | --- | --- | --- | --- | --- | --- | --- | --- | --- |
|  | **Aorta Calcium Score n=81** | | **Coronary Calcium Score n=81** | | **CT Score n=99** | | **Max-IMT (mm) n=136** | | **LV Mass Index n=203** | | **Pulse Wave Velocity (m/sec) n=185** | |
| **Measures** | **r** | **p-value** | **r** | **p-value** | **r** | **p-value** | **r** | **p-value** | **r** | **p-value** | **r** | **p-value** |
| **Total HDL Particles (µmol/L)** | 0.03 | 0.77 | -0.03 | 0.76 | -0.01 | 0.94 | 0.04 | 0.64 | -0.12 | 0.08 | 0.01 | 0.88 |
| **Large HDL (µmol/L)** | 0.08 | 0.50 | -0.15 | 0.18 | -0.17 | 0.10 | 0.06 | 0.45 | 0.03 | 0.62 | 0.07 | 0.36 |
| **Medium HDL (µmol/L)** | 0.04 | 0.72 | -0.03 | 0.78 | -0.01 | 0.91 | 0.00 | 0.98 | -0.15* | 0.04* | 0.04 | 0.60 |
| **Small HDL (µmol/L)** | -0.03 | 0.78 | 0.07 | 0.54 | 0.09 | 0.36 | 0.01 | 0.95 | -0.06 | 0.36 | -0.05 | 0.48 |
| **HDL Size (nm)** | 0.07 | 0.51 | -0.11 | 0.31 | -0.12 | 0.22 | 0.06 | 0.49 | 0.11 | 0.13 | 0.07 | 0.35 |
| **HDL cholesterol (mg/dL)** | 0.07 | 0.53 | -0.14 | 0.20 | -0.13 | 0.19 | 0.04 | 0.63 | -0.01 | 0.89 | 0.07 | 0.32 |
| CT, cardiac computed tomography; IMT, intimal-medial thickness; LV, left ventricle; HDL, high-density lipoprotein | | | | | | | | | | | | |
