# Supplementary material for: High density lipoprotein particle size and function associate with new cardiovascular events in patients with chronic kidney disease
Source: PLoS One. 2025 Apr 1;20(4):e0320803. doi: 10.1371/journal.pone.0320803 (PMC11960887; doi:10.1371/journal.pone.0320803)
Supplement: S4 Table — Hazard ratios, 95% confidence intervals, and p-values are displayed. Statistically significant (p < 0.05) hazard ratios and p-values are bolded. (DOCX) [file pone.0320803.s004.docx]

| **S4 Table. Cox proportional hazards model for time to the new cardiovascular event by significant high density lipoprotein measures.** Hazard ratios, 95% confidence intervals, and p-values are displayed. Statistically significant (p<0.05) hazard ratios and p-values are bolded. | | | | | | | | | | | | | | | | | |  |  |
| --- | --- | --- | --- | --- | --- | --- | --- | --- | --- | --- | --- | --- | --- | --- | --- | --- | --- | --- | --- |
|  | **Model 1** | | **Model 2** | | **Model 3** | | **Model 4** | | **Model 5** | | **Model 6** | | **Model 7** | | **Model 8** | |  |  |  |
|  | *Univariable model****** | | *Measure, age, race, gender, prior CVD history* | | *Measure, age, prior CVD history, diabetes, SBP* | | *Measure, age, prior CVD history, diabetes, eGFR* | | *Measure, age, prior CVD history, diabetes, UPCR* | | *Measure, age, prior CVD history, diabetes, HDL* | | *Measure, age, prior CVD history, diabetes, LDL* | | *Measure, age, prior CVD history, diabetes, statin* | |  |  |  |
|  |  |  |  |  |  |  |  |  |  |  |  |  |  |  |  |  |  | |  |
|  |  |  |  |  |  |  |  |  |  |  |  |  |  |  |  |  |  | |  |
| **Measures** | **HR** | **p-value** | **HR** | **p-value** | **HR** | **p-value** | **HR** | **p-value** | **HR** | **p-value** | **HR** | **p-value** | **HR** | **p-value** | **HR** | **p-value** |  | |  |
|  | **(95% CI)** |  | **(95% CI)** |  | **(95% CI)** |  | **(95% CI)** |  | **(95% CI)** |  | **(95% CI)** |  | **(95% CI)** |  | **(95% CI)** |  |  | |  |
| **Total HDL Particles** | 0.96 | 0.17 | 0.95 | 0.14 | 0.99 | 0.77 | 1.00 | 0.87 | 0.99 | 0.67 | 0.94 | 0.08 | 0.99 | 0.79 | 0.979 | 0.49 |  | |  |
| **(µmol/L)** | (0.90, 1.02) |  | (0.89, 1.02) |  | (0.93, 1.05) |  | (0.94, 1.06) |  | (0.93, 1.05) |  | (0.88, 1.01) |  | (0.93, 1.05) |  | (0.92, 1.04) |  |  | |  |
| **Large HDL** | **1.10** | **0.04** | **1.15** | **0.01** | **1.20** | **<.01** | **1.21** | **<.01** | **1.20** | **<.01** | **1.27** | **0.01** | **1.19** | **<.01** | **1.216** | **<.01** |  | |  |
| **(µmol/L)** | **(1.01, 1.19)** |  | **(1.03, 1.27)** |  | **(1.09, 1.33)** |  | **(1.09, 1.34)** |  | **(1.08, 1.32)** |  | **(1.07, 1.50)** |  | **(1.07, 1.33)** |  | **(1.10, 1.35)** |  |  | |  |
| **Medium HDL** | 0.91 | 0.13 | 0.90 | 0.12 | 0.92 | 0.18 | 0.92 | 0.20 | 0.92 | 0.21 | 0.94 | 0.30 | 0.93 | 0.27 | 0.914 | 0.18 |  | |  |
| **(µmol/L)** | (0.80, 1.03) |  | (0.78, 1.03) |  | (0.81, 1.04) |  | (0.81, 1.05) |  | (0.81, 1.05) |  | (0.83, 1.06) |  | (0.82, 1.06) |  | (0.80, 1.04) |  |  | |  |
| **Small HDL** | 0.95 | 0.08 | 0.94 | 0.06 | 0.96 | 0.19 | 0.96 | 0.21 | 0.95 | 0.13 | 0.94 | 0.05 | 0.96 | 0.23 | 0.945 | 0.08 |  | |  |
| **(µmol/L)** | (0.89, 1.01) |  | (0.89, 1.00) |  | (0.90, 1.02) |  | (0.91, 1.02) |  | (0.90, 1.01) |  | (0.89, 1.00) |  | (0.91, 1.02) |  | (0.89, 1.01) |  |  | |  |
| **HDL Size** | **2.09** | **0.01** | **2.68** | **<.01** | **3.05** | **<.01** | **3.08** | **<.01** | **3.35** | **<.01** | **3.23** | **<.01** | **2.57** | **<.01** | **3.675** | **<.01** |  | |  |
| **(nm)** | **(1.24, 3.52)** |  | **(1.47, 4.90)** |  | **(1.69, 5.53)** |  | **(1.70, 5.59)** |  | **(1.81, 6.22)** |  | **(1.52, 6.84)** |  | **(1.38, 4.79)** |  | **(1.91, 7.08)** |  |  | |  |
| **HDL cholesterol** | 1.02 | 0.18 | 1.03 | 0.08 | **1.04** | **<.01** | **1.04** | **<.01** | **1.04** | **<.01** | **1.09** | **0.03** | **1.03** | **0.01** | **1.041** | **<.01** |  | |  |
| **(mg/dL)** | (0.99, 1.04) |  | (1.00, 1.05) |  | **(1.01, 1.06)** |  | **(1.02, 1.07)** |  | **(1.01, 1.06)** |  | **(1.01, 1.18)** |  | **(1.01, 1.06)** |  | **(1.01, 1.07)** |  |  | |  |
| *Model 1 is a univariable model with each separate HDL measure, while Models 2-8 represent multivariable models of the respective HDL measures with additional covariates. | | | | | | | | | | | | | | | | | |  | |
| CVD, cardiovascular disease; SBP, systolic blood pressure; UPCR, urine protein creatinine ratio; HDL, high-density lipoprotein; LDL, low-density lipoprotein; HR, hazard ratio; CI, confidence interval. | | | | | | | | | | | | | | | | | |  | |
