# Supplementary material for: High density lipoprotein particle size and function associate with new cardiovascular events in patients with chronic kidney disease
Source: PLoS One. 2025 Apr 1;20(4):e0320803. doi: 10.1371/journal.pone.0320803 (PMC11960887; doi:10.1371/journal.pone.0320803)
Supplement: S5 Table — Correlation coefficients (r) and corresponding raw p-values are given; significant P-values < 0.05 are indicated with an asterisk *, and those that pass significance after false discovery rate correction are bolded. N = 242. (DOCX) [file pone.0320803.s005.docx]

| **S5 Table. Relationship of high-density lipoprotein measures to cholesterol efflux capacity.** Correlation coefficients (r) and corresponding raw p-values are given; significant P-values <0.05 are indicated with an asterisk*, and those that pass significance after false discovery rate correction are bolded. N=242. | | |
| --- | --- | --- |
|  | **CEC** | |
| **Measures** | **r** | **p-value** |
| **Total HDL Particles (µmol/L)** | **0.23** | ***<.01*** |
| **Large HDL (µmol/L)** | **0.34** | ***<.01*** |
| **Medium HDL (µmol/L)** | 0.01 | 0.94 |
| **Small HDL (µmol/L)** | 0.04 | 0.59 |
| **HDL Size (nm)** | **0.26** | ***<.01*** |
| **HDL cholesterol (mg/dL)** | **0.37** | ***<.01*** |
| HDL, high-density lipoprotein; CEC, cholesterol efflux capacity | | |
