# Supplementary material for: High density lipoprotein particle size and function associate with new cardiovascular events in patients with chronic kidney disease
Source: PLoS One. 2025 Apr 1;20(4):e0320803. doi: 10.1371/journal.pone.0320803 (PMC11960887; doi:10.1371/journal.pone.0320803)
Supplement: S6 Table — Correlation coefficients (r) and corresponding raw p-values are given; significant P-values < 0.05 are indicated with an asterisk *, and those that pass significance after false discovery rate correction are bolded. N = 242. (DOCX) [file pone.0320803.s006.docx]

| **S6 Table. Relationship of high density lipoprotein measures to HDL oxidation measures.** Correlation coefficients (r) and corresponding raw p-values are given; significant P-values <0.05 are indicated with an asterisk*, and those that pass significance after false discovery rate correction are bolded. N=242. | | | | | | |
| --- | --- | --- | --- | --- | --- | --- |
|  | **3-chlorotyrosine^1^** | | **o,o'-dityrosine^1^** | | **3-nitrotyrosine^1^** | |
| **Measures** | **r** | **p-value** | **r** | **p-value** | **r** | **p-value** |
| **Total HDL Particles (µmol/L)** | 0.08 | 0.23 | 0.12 | 0.06 | 0.04 | 0.52 |
| **Large HDL (µmol/L)** | -0.03 | 0.61 | 0.01 | 0.86 | 0.06 | 0.34 |
| **Medium HDL (µmol/L)** | 0.12 | 0.06 | 0.07 | 0.27 | 0.02 | 0.76 |
| **Small HDL (µmol/L)** | 0.03 | 0.64 | 0.08 | 0.24 | -0.01 | 0.93 |
| **HDL Size (nm)** | -0.05 | 0.40 | -0.05 | 0.42 | 0.03 | 0.70 |
| **HDL Cholesterol (mg/dL)** | 0.04 | 0.54 | 0.06 | 0.39 | 0.07 | 0.31 |
| **CEC (%)** | 0.05 | 0.42 | ***0.17*** | ***<.01*** | -0.01 | 0.87 |
| HDL, high-density lipoprotein; CEC, cholesterol efflux capacity ^1^Represents units (µM/mM tyrosine) | | | | | | |
