# Supplementary material for: High density lipoprotein particle size and function associate with new cardiovascular events in patients with chronic kidney disease
Source: PLoS One. 2025 Apr 1;20(4):e0320803. doi: 10.1371/journal.pone.0320803 (PMC11960887; doi:10.1371/journal.pone.0320803)
Supplement: S7 Table — Correlation coefficients (r) and corresponding raw p-values are given; significant P-values < 0.05 are indicated with an asterisk *, and those that pass significance after false discovery rate correction are bolded. N = 325. (DOCX) [file pone.0320803.s007.docx]

| **S7 Table. Correlation of cholesterol efflux capacity and high-density lipoprotein oxidation measures with clinical measures.** Correlation coefficients (r) and corresponding raw p-values are given; significant P-values <0.05 are indicated with an asterisk*, and those that pass significance after false discovery rate correction are bolded. N=325. | | | | | | | | | | | | | | | | | | | |
| --- | --- | --- | --- | --- | --- | --- | --- | --- | --- | --- | --- | --- | --- | --- | --- | --- | --- | --- | --- |
|  | **Blood Pressure (mmHg)** | | **CRP (mg/dL)** | | **eGFR (ml/min)** | | **A/C ratio (g/g creatinine)** | | **Serum Albumin (g/dL)** | | **Cholesterol (mg/dL)** | | | **HDL (mg/dL)** | | | **LDL (mg/dL)** | | |
| **Measures** | **r** | **p-value** | **r** | **p-value** | **r** | **p-value** | **r** | **p-value** | **r** | **p-value** | **r** | **p-value** | **r** | | **p-value** | **r** | | **p-value** |  |
| **3-chlorotyrosine^1^** | -0.01 | 0.81 | 0.00 | 0.93 | 0.07 | 0.22 | -0.11 | 0.06 | 0.09 | 0.13 | *-0.12** | *0.04** | 0.02 | | 0.71 | *-0.15** | | *0.01** |  |
| **o,o'-dityrosine^1^** | 0.00 | 0.98 | -0.04 | 0.45 | 0.10 | 0.07 | -0.05 | 0.38 | 0.01 | 0.84 | -0.04 | 0.44 | *0.11** | | *0.04** | -0.11 | | 0.05 |  |
| **3-nitrotyrosine^1^** | -0.05 | 0.36 | 0.05 | 0.34 | 0.04 | 0.45 | 0.06 | 0.26 | -0.09 | 0.11 | 0.00 | 0.96 | 0.01 | | 0.81 | -0.01 | | 0.84 |  |
| **CEC (%)** | -0.11 | 0.05 | -0.02 | 0.76 | -0.02 | 0.72 | -0.09 | 0.13 | 0.03 | 0.60 | 0.05 | 0.38 | ***0.31*** | | ***<.01*** | -0.04 | | 0.49 |  |
| eGFR, estimated glomerular filtration rate; HDL, high-density lipoprotein; LDL, low-density lipoprotein; A/C, albumin to creatinine; CRP, C-reactive protein; CEC, cholesterol efflux capacity | | | | | | | | | | | | | | | | | | | |
| ^1^µM/mM tyrosine | | | | | | | | | | | | | | | | | | | |
