# Supplementary material for: High density lipoprotein particle size and function associate with new cardiovascular events in patients with chronic kidney disease
Source: PLoS One. 2025 Apr 1;20(4):e0320803. doi: 10.1371/journal.pone.0320803 (PMC11960887; doi:10.1371/journal.pone.0320803)
Supplement: S8 Table — Correlation coefficients (r) and corresponding raw p-values are given; significant P-values < 0.05 are indicated with an asterisk *, and those that pass significance after false discovery rate correction are bolded. (DOCX) [file pone.0320803.s008.docx]

| **S8 Table. Relationship of lipoprotein oxidation measures with subclinical markers of cardiovascular disease.** Correlation coefficients (r) and corresponding raw p-values are given; significant P-values <0.05 are indicated with an asterisk*, and those that pass significance after false discovery rate correction are bolded. | | | | | | | | |
| --- | --- | --- | --- | --- | --- | --- | --- | --- |
|  | **3-chlorotyrosine^1^** | | ***o,o'*-dityrosine^1^** | | **3-nitrotyrosine^1^** | | **CEC (%)** | |
| **Measures** | **r** | **p-value** | **r** | **p-value** | **r** | **p-value** | **r** | **p-value** |
| **Aorta Calcium Score (n=84)** | -0.06 | 0.62 | -0.04 | 0.75 | -0.21 | 0.05 | 0.07 | 0.53 |
| **Coronary Calcium Score (n=84)** | -0.09 | 0.44 | -0.12 | 0.29 | -0.16 | 0.15 | -0.02 | 0.83 |
| **CT Score (n=99)** | -0.03 | 0.77 | -0.11 | 0.20 | -0.21* | 0.02* | -0.05 | 0.58 |
| **Max IMT (mm) (n=182)** | -0.10 | 0.18 | -0.02 | 0.77 | 0.01 | 0.94 | 0.01 | 0.92 |
| **LV Mass Index (n=211)** | -0.02 | 0.73 | -0.10 | 0.17 | -0.04 | 0.55 | -0.05 | 0.48 |
| **Pulse Wave Velocity (m/sec) (n=251)** | -0.01 | 0.92 | 0.03 | 0.69 | 0.01 | 0.82 | -0.02 | 0.79 |
| CEC, cholesterol efflux capacity; CT, cardiac computed tomography; IMT, intimal-medial thickness; LV, left ventricle | | | | | | | | |
| ^1^Represent units (µM/mM tyrosine) | | | | | | | | |
