# Supplementary material for: High density lipoprotein particle size and function associate with new cardiovascular events in patients with chronic kidney disease
Source: PLoS One. 2025 Apr 1;20(4):e0320803. doi: 10.1371/journal.pone.0320803 (PMC11960887; doi:10.1371/journal.pone.0320803)
Supplement: S9 Table — Hazard ratios, 95% confidence intervals, and p-values are displayed. Statistically significant (p < 0.05) hazard ratios and p-values are bolded. (DOCX) [file pone.0320803.s009.docx]

| **S9 Table. Cox proportional hazards model for time to new cardiovascular event by significant lipoprotein oxidation measures.** Hazard ratios, 95% confidence intervals, and p-values are displayed. Statistically significant (p<0.05) hazard ratios and p-values are bolded. | | | | | | | | | | | | | | | | | | |  |
| --- | --- | --- | --- | --- | --- | --- | --- | --- | --- | --- | --- | --- | --- | --- | --- | --- | --- | --- | --- |
|  | **Model 1** | | **Model 2** | | **Model 3** | | **Model 4** | | **Model 5** | | **Model 6** | | | **Model 7** | | | **Model 8** | |  |
|  | *Univariable model* | | *Measure, age, race, gender, prior CVD history* | | *Measure, age, prior CVD history, diabetes, SBP* | | *Measure, age, prior CVD history, diabetes, eGFR* | | *Measure, age, prior CVD history, diabetes, ACR* | | *Measure, age, prior CVD history, diabetes, HDL* | | | *Measure, age, prior CVD history, diabetes, LDL* | | | *Measure, age, prior CVD history, diabetes, statin* | |  |
|  |  |  |  |  |  |  |  |  |  |  |  |  |  |  |  |  |  |  |  |
|  |  |  |  |  |  |  |  |  |  |  |  |  |  |  |  |  |  |  |  |
| **Measures*** | **HR** | **p-value** | **HR** | **p-value** | **HR** | **p-value** | **HR** | **p-value** | **HR** | **p-value** | **HR** | **p-value** | **HR** | | **p-value** | **HR** | | **p-value** |  |
|  | **(95% CI)** |  | **(95% CI)** |  | **(95% CI)** |  | **(95% CI)** |  | **(95% CI)** |  | **(95% CI)** |  | **(95% CI)** | |  | **(95% CI)** | |  |  |
| **3-chlorotyrosine^1^** | 1.01 | 0.78 | 1.02 | 0.55 | 1.02 | 0.55 | 1.02 | 0.51 | 1.02 | 0.53 | 1.01 | 0.61 | 1.01 | | 0.75 | 1.021 | | 0.43 |  |
|  | (0.957, 1.06) |  | (0.965, 1.069) |  | (0.966, 1.066) |  | (0.967, 1.07) |  | (0.966, 1.069) |  | (0.963, 1.066) |  | (0.96, 1.059) | |  | (0.97, 1.074) | |  |  |
| **3-nitrotyrosine^1^** | 0.98 | 0.39 | 0.99 | 0.58 | 0.98 | 0.37 | 0.98 | 0.54 | 0.98 | 0.37 | 0.98 | 0.38 | 0.99 | | 0.60 | 0.988 | | 0.63 |  |
|  | (0.931, 1.029) |  | (0.937, 1.037) |  | (0.93, 1.027) |  | (0.936, 1.035) |  | (0.927, 1.028) |  | (0.929, 1.028) |  | (0.939, 1.037) | |  | (0.939, 1.039) | |  |  |
| **o,o'-dityrosine^1^** | 0.98 | 0.30 | 0.98 | 0.45 | 0.98 | 0.41 | 0.98 | 0.48 | 0.98 | 0.45 | 0.97 | 0.26 | 0.97 | | 0.26 | 0.984 | | 0.52 |  |
|  | (0.929, 1.023) |  | (0.935, 1.03) |  | (0.936, 1.028) |  | (0.937, 1.031) |  | (0.936, 1.03) |  | (0.929, 1.02) |  | (0.928, 1.02) | |  | (0.937, 1.034) | |  |  |
| *Model 1 is a univariable model with each separate HDL measure, while Models 2-8 represent multivariable models of the respective HDL measures with additional covariates. | | | | | | | | | | | | | | | | | | |  |
| CVD, cardiovascular disease; SBP, systolic blood pressure; ACR, albumin to creatinine ratio; HDL, high-density lipoprotein; LDL, low-density lipoprotein; HR, hazard ratio; CI, confidence interval | | | | | | | | | | | | | | | | | | |  |
| ^1^Represent untis (µM/mM tyrosine) | | | | | | | | | | | | | | | | | | |  |
